# Electrophysiology and Behavioral Assessment of the New Molecule SMe1EC2M3 as a Representative of the Future Class of Triple Reuptake Inhibitors

**DOI:** 10.3390/molecules24234218

**Published:** 2019-11-20

**Authors:** Romana Koprdova, Kristina Csatlosova, Barbora Durisova, Eszter Bogi, Magdalena Majekova, Eliyahu Dremencov, Mojmir Mach

**Affiliations:** 1 Institute of Experimental Pharmacology and Toxicology, Centre of Experimental Medicine of the Slovak Academy of Sciences, Dúbravská cesta 9, 841 04 Bratislava, Slovakia; romana.koprdova@savba.sk (R.K.);; 2 Institute of Molecular Physiology and Genetics, Center of Biosciences, Slovak Academy of Sciences, Dúbravská cesta 9, 840 05 Bratislava, Slovakia; 3 Institute of Experimental Endocrinology, Biomedical Research Center, Slovak Academy of Sciences, Dúbravská cesta 9, 845 05 Bratislava, Slovakia

**Keywords:** pyridoindole derivatives, behavior, electrophysiology, triple reuptake inhibitor, rat

## Abstract

SMe1EC2M3 is a pyridoindole derivative related to the neuroleptic drug carbidine. Based on the structural similarities of SMe1EC2M3 and known serotonin (5-HT), norepinephrine, and dopamine reuptake inhibitors, we hypothesized that this compound may also have triple reuptake inhibition efficacy and an antidepressant-like effect. PreADMET and Dragon software was used for in silico prediction of pharmacokinetics and pharmacodynamics of SMe1EC2M3. Forced swim test was used to evaluate its antidepressant-like effects. Extracellular in vivo electrophysiology was used to assess 5-HT, norepinephrine, and dopamine reuptake inhibition efficacy of SMe1EC2M3. PreADMET predicted reasonable intestinal absorption, plasma protein binding, and blood-brain permeability for SMe1EC2M3. Dragon forecasted its efficiency as an antidepressant. Using behavioral measurements, it was found that SMe1EC2M3 decreased immobility time and increase swimming time during the forced swim test (FST). Electrophysiological investigations showed that SMe1EC2M3 dose-dependently suppressed the excitability of 5-HT neurons of the dorsal raphe nucleus (DRN), norepinephrine neurons of the locus coeruleus (LC), and dopamine neurons of the ventral tegmental area (VTA). The SMe1EC2M3-induced suppression of 5-HT, norepinephrine, and dopamine neurons was reversed by the antagonists of serotonin-1A (5-HT_1A_; WAY100135), α-2 adrenergic (α_2_, yohimbine), and dopamine-2 receptors (D_2_, haloperidol), respectively. We conclude that SMe1EC2M3 is prospective triple 5-HT, norepinephrine, and dopamine reuptake inhibitor with antidepressant-like properties, however future studies should be performed to complete the pharmacological profiling of this compound.

## 1. Introduction

Major depression is considered the most severe mental disorder and one of the most severe medical conditions in general, in terms of cost, prevalence, disability, and mortality rate [1]. Although the etiology of depression is not yet completely understood, it is well established that serotonin (5-HT), norepinephrine and dopamine systems play a key role in the treatment of this disorder [2]. 

Selective serotonin (5-HT) reuptake inhibitors (SSRIs) have been used as first-choice drugs for the treatment of depression, indicating the key role of 5-HT neurotransmission in pharmacotherapy of depression. However, the clinical efficacy of SSRIs remains limited. The remission of the symptoms happens only in 30–40% of the patients after the treatment with a first-choice SSRI [3]. Even if patients meet remission criteria, at least one symptom remains unresolved in almost all patients. These residual symptoms possess a high risk to relapse, chronic course, and even suicide [4].

Brain norepinephrine system is involved in attention modulation, arousal, and cognitive performances during various behavioral tasks. Since depression is often manifested with fatigue, reduced general activity, and loss of concentration [5], boosting brain norepinephrine transmission might be beneficial in the treatment of this disorder. It was indeed reported that the dual 5-HT and norepinephrine reuptake inhibitors (SNRIs) have lower relapse rates than SSRIs, suggesting that the simultaneous stimulation of 5-HT and norepinephrine neurotransmission has higher efficacy in the treatment of depression than the solo stimulation of 5-HT tone [3]. 

Mesocorticolimbic dopamine system is fundamental in motivation, reward, and reinforcement [6]. Since these behavioral features are impaired in depression [5], it is likely that the stimulation of dopamine transmission will contribute to the successful treatment of depression [7]. It was therefore proposed that triple 5-HT, norepinephrine and dopamine reuptake inhibitors will have higher clinical efficacy than SSRIs and SNRIs [8]. Several triple reuptake inhibitors (TRIs) are now under different stages of preclinical and clinical investigations, such as SEP225289 [9,10], DOV216303 [10], and NS18283 [11]. 

**Figure 1:**
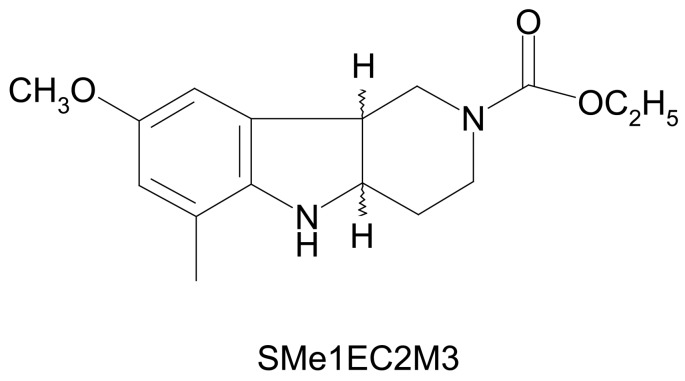
Structural formula of ((±)-*cis* ethyl 8-methoxy-6-methyl-3,4,4a,5,9b*H*-hexahydro-1*H*-pyrido[4,3-b]indole-2-carboxylate (SMe1EC2M3).

In our study we focused on SMe1EC2M3 ((±)-*cis* ethyl 8-methoxy-6-methyl-3,4,4a,5,9b*H*-hexahydro-1*H*-pyrido[4,3-b]indole-2-carboxylate (Figure 1). It is one of 82 pyridoindole derivatives designed and synthesized at the Institute of Experimental Pharmacology and Toxicology of Slovak Academy of Sciences [12]. Although the original aim was to develop an antioxidant drug for the treatment of brain injury, the possibility of an antidepressant-like effect also came in question because of SMe1EC2M3 is structurally related to carbolines and pyridoindoles, known for their neuropleptic and antidepressant properties. β-carbolines and γ-carbolines came into attention of anti-neurodegenerative drug designers due to the assorted activities of *harmala* alkaloids (harmaline, harmine, harmane, etc.), which are β-carbolines, as well as by introducing the synthetic compounds with γ-carboline scaffold in pre-clinical and clinical research: dimebon (latrepirdine), karbidine, stobadine, flutroline [13,14,15]. As an indole scaffold occurs in the structure of known TRIs, such as (3a,7a)-3a-(3,4-dichlorophenyl)-2-methyloctahydro-1*H*-isoindole and (3aS,6aR)-3a-(3,4-dichlorophenyl)-2-methyloctahydrocyclopenta[c]pyrrole [16], we decided to test antidepressant-like properties based on 5-HT, norepinephrine, and dopamine reuptake inhibition.

In this study, we used PreADMET (Yonsei University, Yonsei, Republic of Korea) software to assess basic pharmacokinetics of SMe1EC2M3, and Dragon (Talete Srl., Milano, Italy) to forecast its antidepressant efficacy. We employed forced swim test (FST) in order to investigate the antidepressant-like properties of SMe1EC2M3. In vivo electrophysiology was used to assess the efficacy of SMe1EC2M3 as a blocker of 5-HT (SERT), norepinephrine (NET) and dopamine transporters (DAT).

## 2. Results

### 2.1. In Silico Study

The in silico prediction results are summarized in Table 1. According to the ADMET predictions, compound SMe1EC2M3 penetrates into the brain, has good human intestinal absorption and reasonable binding to plasma proteins. The partition and distribution coefficients speak for a good bioavailability. The compound fulfills the Lipinski rules of five, which favors it as a drug candidate. The BLTF96 value was in the range of low toxic compounds [17]. According to the prediction of therapeutic properties, we could expect antidepressant, but not antipsychotic and hypnotic effects.

### 2.2. Behavioral Experiments

**Figure 2:**
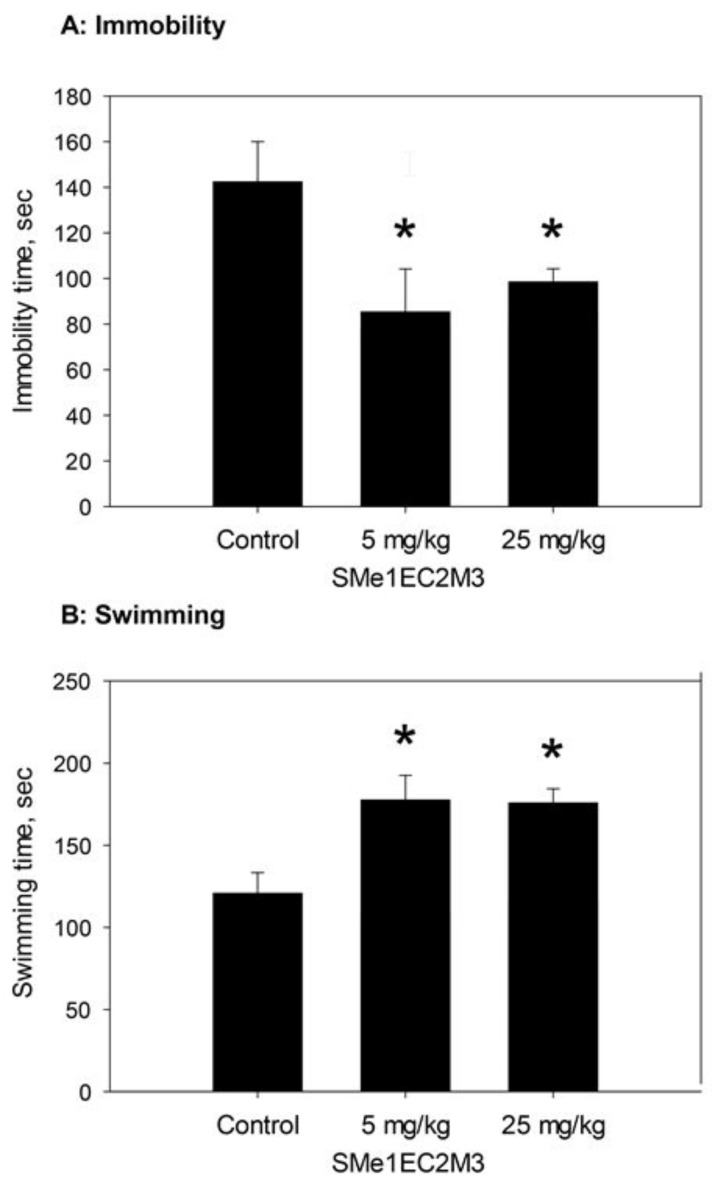
Effects of SMe1EC2M3 on the behavioral characteristics of Wistar rats. (**A**): immobility time during the FST test; (**B**): swimming time during the FST; * *p* < 0.05, Fisher´s LSD post-hoc test.

Figure 2 shows the effect of SMe1EC2M3 (5 and 25 mg/kg) on the time of immobility during the FST (A) and time of swimming in FST (B). 

SMe1EC2M3 significantly decreased the rats’ immobility time during the FST. The main effect of treatment was significant (F_2,26_ = 4.1725, p = 0.027, n = 10 rats/group), and the following Fisher’s LSD post-hoc test revealed significant decrease of immobility time in both doses of SMe1EC2M3 compared to controls (*p* < 0.05). SMe1EC2M3 also significantly increased the rats’ swimming time. The main effect of treatment was significant (F_2,26_ = 6.7893, p = 0.004, n = 10 rats/group), following Fisher´s LSD post-hoc test revealed significant increase of swimming time in both doses of SMe1EC2M3 compared to controls (*p* < 0.05). We did not find significant differences in time spent of climbing in FST.

### 2.3. Electrophysiological Experiments

The basal activities of 5-HT, norepinephrine, and dopamine neurons were 2.30 ± 0.51, 4.00 ± 1.00, and 7.67 ± 1.56 Hz, respectively. 

**Figure 3:**
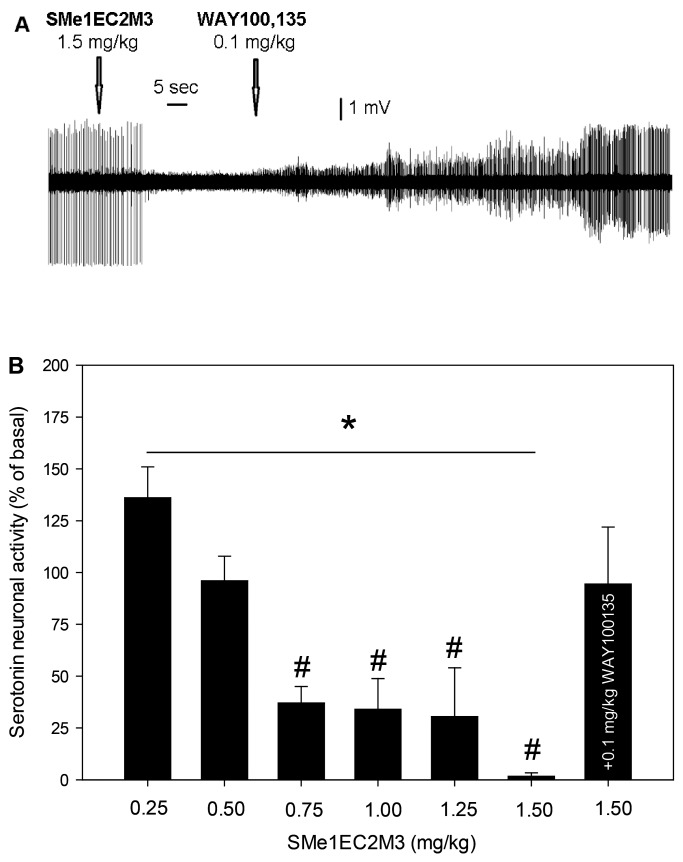
Effects of SMe1EC2M3 and WAY100135 on the excitability of 5-HT neurons of the DRN. (**A**): representative recording from a single neuron; (**B**): summary effect from seven neurons from seven rats, expressed as % ± SEM of basal activity. SMe1EC2M3 was applied at the cumulative doses of 0.25–1.5 mg/kg (i.v.). Two minutes after the last SMe1EC2M3 administration, a selective antagonist of 5-HT_1A_ receptors, WAY100135, was injected (0.1 mg/kg, i.v.); *p < 0.05, one-way ANOVA for repeated measures; #*p* < 0.05, Fisher´s LSD post-hoc test.

Figure 3 shows the effect of SMe1EC2M3 and WAY100135 on the excitability of 5-HT neurons of the DRN. SMe1EC2M3 dose-dependently suppressed the firing activity of 5-HT neurons, with the maximal 98 ± 2%-inhibition observed after the administration of 1.5 mg/kg of SMe1EC2M3. Subsequent administration of WAY100135 reversed SMe1EC2M3-induced inhibition of 5-HT neurons to the values statistically indistinguishable from the baseline (95 ± 28%). One-way ANOVA for repeated measures revealed a significant effect of time (F_7,29_ = 5.51, *p* < 0.05, n = 7 neurons from seven rats). Fisher´s LSD post-hoc test confirmed a significant decrease in the excitability of 5-HT neurons after the administration of 0.75–1.5 mg/kg of SMe1EC2M3 (*p* < 0.05).

**Figure 4:**
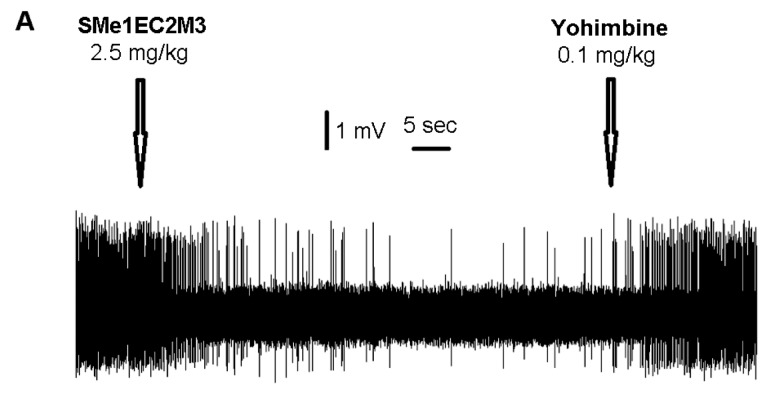

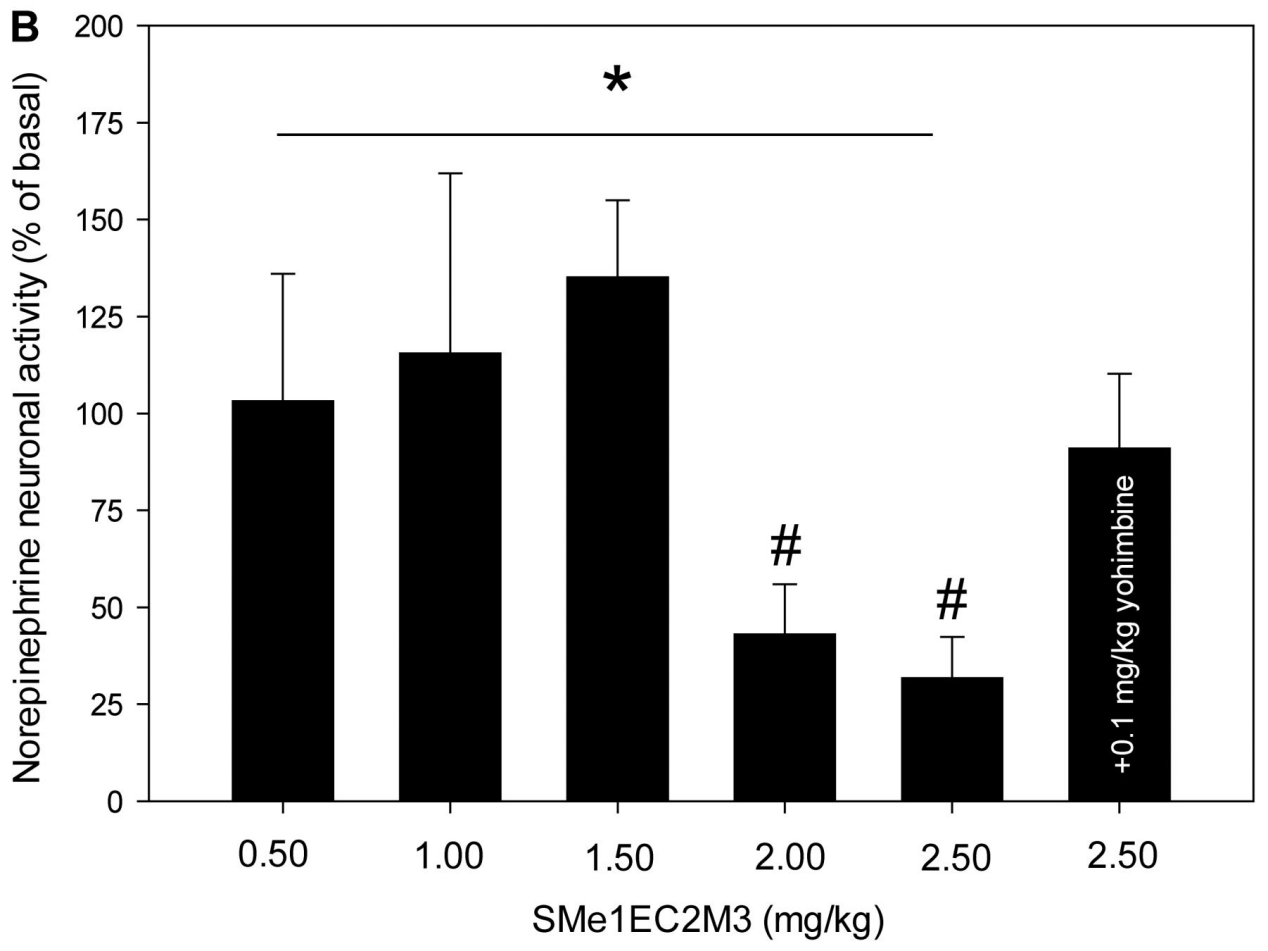
Effect of SMe1EC2M3 on the excitability of norepinephrine neurons of the locus coeruleus. (**A**): representative recording from a single neuron; (**B**): summary result from seven neurons from seven rats, expressed as % ± SEM of basal activity. SMe1EC2M3 was applied at the cumulative doses of 0.5–2.5 mg/kg (i.v.). Two minutes after the last SMe1EC2M3 administration, a selective antagonist of α_2_ adrenoceptors, yohimbine, was injected (0.1 mg/kg, i.v.); *p < 0.05, one-way ANOVA for repeated measures; #*p* < 0.05, Fisher´s LSD post-hoc test.

Figure 4 shows the effect of SMe1EC2M3 and yohimbine on the excitability of norepinephrine neurons of the LC. SMe1EC2M3 suppressed the firing activity of norepinephrine neurons, with the maximal 68 ± 11%-inhibition observed after the administration of 2.5 mg/kg of SMe1EC2M3. Subsequent administration of yohimbine reversed the SMe1EC2M3-induced inhibition of norepinephrine neurons to the values statistically indistinguishable from the baseline (91 ± 20%). One-way ANOVA for repeated measures revealed a significant effect of time (F_6,36_ = 3.33, *p* < 0.05, n = 7 neurons from seven rats). Fisher´s LSD post-hoc test confirmed a significant decrease in the excitability of norepinephrine neurons after the administration of 2.0–2.5 mg/kg of SMe1EC2M3 (*p* < 0.05).

**Figure 5:**
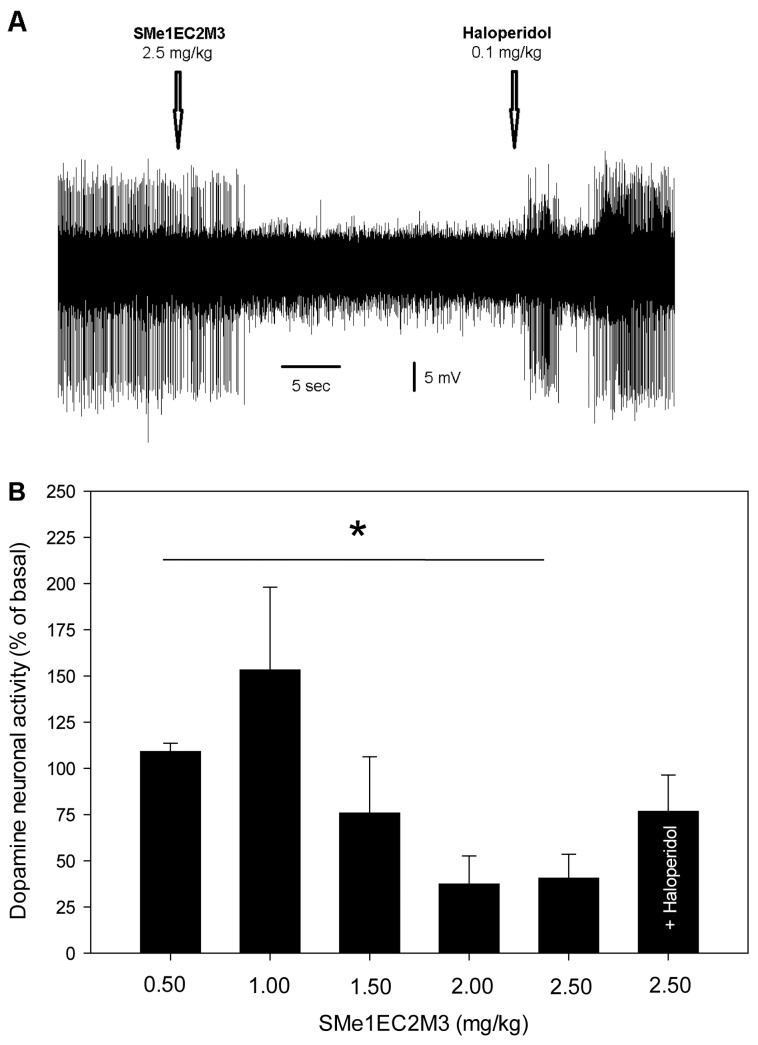
Effect of SMe1EC2M3 on the excitability of dopamine neurons of the ventral tegmental area. (**A**): representative recording from a single neuron; (**B**): summary result from seven neurons from seven rats, expressed as % ± SEM of basal activity. SMe1EC2M3 was applied at the cumulative doses of 0.5–2.5 mg/kg (i.v.). Two minutes after the last SMe1EC2M3 administration, a selective antagonist of D_2_ receptors, haloperidol, was injected (0.1 mg/kg, i.v.); *p < 0.05, one-way ANOVA for repeated measures.

Figure 5 shows the effect of SMe1EC2M3 and haloperidol on the excitability of dopamine neurons of the VTA. SMe1EC2M3 suppressed the firing activity of dopamine neurons, with the maximal 63 ± 15%-inhibition observed after the administration of 2.0 mg/kg of SMe1EC2M3. Subsequent administration of haloperidol reversed SMe1EC2M3-induced to the values statistically indistinguishable from the baseline (78 ± 20%). One-way ANOVA for repeated measures revealed a significant effect of time (F_6,37_ = 2.67, *p* < 0.05, n = 7 neurons from seven rats).

## 3. Discussion

Assessments performed with a PreADMET software predicted good HIA and BBB permeability and reasonable PPB for SMe1EC2M3. Dragon forecasted its low toxicity and potent antidepressant, but not antipsychotic or hypnotic effect. Based on these characteristics, SMe1EC2M3 was chosen for the further behavioral and electrophysiological studies in vivo. In the behavioral assessments, SMe1EC2M3 diminished the immobility time during the FST and increased swimming time without affecting climbing. Physiologically, SMe1EC2M3 inhibited the excitability of 5-HT neurons of the DRN, norepinephrine neurons of the LC, and dopamine neurons of the VTA. Subsequent administration of the antagonist of corresponding autoreceptors (5-HT_1A_ antagonist WAY100135, α_2_-adrenergic antagonist yohimbine, and D_2_ antagonist haloperidol) reversed the SMe1EC2M3-induced inhibition of 5-HT, norepinephrine, and dopamine neurons.

The present study showed that SMe1EC2M3 reduced the immobility of the rats (Figure 2A) and prolonged their swimming time during FST (Figure 2B). FST is a well-established behavioral test designated to predict the antidepressant-like properties of the drugs in rodents [18,19]. The antidepressant-like effect of SMe1EC2M3, assessed by the immobility time during the FST, is comparable to that of the SSRI escitalopram [20], SNRI venlafaxine [21], and TRIs such as DOV216303 [22], JZAD-IV-22 [23] and LPM570065 [24]. Climbing behavior (which is affected by the escapability of the situation) is increased by noradrenergic drugs, without affecting swimming, and SSRIs increase swimming without affecting climbing [25,26,27,28]. There are numerous physiological mechanisms by which NE and 5-HT neurotransmission might interact to influence the reciprocal neurotransmitter system. SNRIs milnacipran, duloxetine and venlafaxine, are effective in the FST, but only venlafaxine demonstrate a pattern where both active behaviors are increased in the FST. Drugs, which combine effects on different neurotransmitter systems, especially DA and 5-HT systems, are able to evoke multiple active response patterns in the FST [29]. None of the studies evaluating the antidepressant-like activity of TRIs has examined the climbing response, to compare for example, their in vivo potency at stimulating brain serotonergic and noradrenergic neurotransmissions [30]. For example, TRIs referred to as PRC025 and PRC050 (racemic analogs of venlafaxine) increased time spent swimming and reduced time spent immobile in the forced swim test [31]. These findings are in concordance with our study.

SMe1EC2M3 dose-dependently inhibited the firing activity of 5-HT neurons of the DRN. The maximal and almost complete suppression of 5-HT excitability was observed after the administration of 1.5 mg/kg of SMe1EC2M3. This inhibition was completely reversed after the subsequent administration of the selective antagonist of 5-HT_1A_ receptors WAY100135. It can be therefore suggested that the SMe1EC2M3-induced inhibition of the excitability of 5-HT neurons is mediated via the blockade of 5-HT reuptake, increase in extracellular 5-HT levels, and subsequent activation of 5-HT_1A_ receptors located on the cell bodies of 5-HT neurons [32,33]. The efficacy of SMe1EC2M3 as a blocker of SERT is comparable to that of the SSRIs citalopram [29] and escitalopram [32,33], SNRI venlafaxine [34], and TRIs such as SEP225289 and DOV216303 [10].

SMe1EC2M3 inhibited the firing activity of norepinephrine neurons of the LC. The maximal inhibition was observed after the administration of 2.5 mg/kg of SMe1EC2M3. This inhibition was completely reversed after the subsequent administration of the selective antagonist of α_2_-adrenoceptors yohimbine. It can be therefore suggested that the SMe1EC2M3-induced inhibition of the excitability of norepinephrine neurons is mediated via the blockade of norepinephrine reuptake, increase in extracellular norepinephrine levels, and subsequent activation of α_2_-adreneregic autoreceptors located on the cell bodies of norepinephrine neurons [34]. The efficacy of SMe1EC2M3 as a blocker of NET is comparable to that of the SNRI venlafaxine [34] and TRIs SEP225289 and DOV216303 [10].

SMe1EC2M3 inhibited the firing activity of dopamine neurons of the VTA. The maximal inhibition was observed after the administration of 2 mg/kg of SMe1EC2M3. This inhibition was reversed after the subsequent administration of the selective antagonist of D_2_ receptors haloperidol. It can be therefore suggested that the SMe1EC2M3-induced inhibition of the excitability of dopamine neurons is mediated via the blockade of dopamine reuptake, increase in extracellular dopamine levels, and subsequent activation of D_2_ autoreceptors located on the cell bodies of dopamine neurons [10]. The efficacy of SMe1EC2M3 as a blocker of DAT is comparable to that of the TRIs SEP225289 and DOV216303 [10].

SMe1EC2M3 almost completely inhibited the firing activity of 5-HT neurons; however, the inhibition of norepinephrine and dopamine excitability was not completed. Similar results were previously obtained with the SNRI venlafaxine [34] and triple reuptake inhibitor SEP225289. DOV216303 did not completely suppressed 5-HT, norepinephrine, or dopamine neurons, but its effect of 5-HT neurons was more robust than on norepinephrine or dopamine ones [10].

The dose of SMe1EC2M3 required for the maximal inhibition of 5-HT neurons (1.5 mg/kg, i.v.) is lower than this required to obtain the highest degree of inhibition of norepinephrine (2.5 mg/kg, i.v.) and dopamine (2.0 mg/kg, i.v.) neurons. It is possible that the affinity of SMe1EC2M3 for the SERT is higher than this for the NET and DAT. The efficacy SMe1EC2M3 for the DAT might be slightly higher than for the NET.

In similarity to the SMe1EC2M3, higher dose of venlafaxine was required to inhibit norepinephrine (3.0 mg/kg, i.v.) than 5-HT neurons (0.4 mg/kg, i.v.) [34]. However, similar dose of SEP225289 was required to obtain the maximal inhibition of 5-HT, norepinephrine, and dopamine neurons (8.0 mg/kg). The dose of DOV216303, required for the maximal inhibition of dopamine neurons (5 mg/kg) was lower than this required to achieve the maximal inhibition of 5-HT and norepinephrine cells (10 mg/kg) [10].

As already mentioned above, SMe1EC2M3 was originally designed, synthetized and tested together with other pyridoindole compounds for their antioxidant properties. In comparison with well-known antioxidant trolox, SMe1EC2M3 showed stronger effect as the inhibitor of lipoperoxidation induced in rat brain homogenate (values of pIC_50_, a negative logarithm of the middle inhibition concentration, were 5.12 for SMe1EC2M3 and 3.93 for trolox [12]. Depression, either diagnosed per se or co-diagnosed with other chronic diseases, is often accompanied with oxidation stress [35,36]. The ability to eliminate the oxidation stress could be an additive property in a multi-target profile of new compound. As the beneficial antioxidant activity of SMe1ECM3 is connected with its ability to scavenge free radicals [37], preserving of the functional molecular structure of this compound is questionable. However, the excellent antioxidant activity of the parent drug stobadine was related also to the possibility to recover itself by natural antioxidants as vitamin E, ascorbate and chromanol [38], which was later supported by the results of in vivo experiments [39]. As the reaction center for the free radical scavenging remained the same for SMe1EC2M3 as it was for stobadine, we can expect a similar behavior of both compounds. Good predicted pharmacokinetic and pharmacodynamical properties mean a plus for SMe1EC2M3 as a promising compound for suppressing depression manifestations.

Summarizing, SMe1EC2M3 displays antidepressant-like properties which were predicted by in silico and subsequently supported by in vivo behavioral studies. In vivo electrophysiological assessments indicate that SMe1EC2M3 might be a potent 5-HT, norepinephrine, and dopamine reuptake inhibitor, with the efficacy comparable to that of SEP225289 and DOV216303. It is also possible that other pyridoindoles exhibit antidepressant-like effect, which is mediated, at least in part, via the 5-HT, norepinephrine, and/or dopamine reuptake inhibition. However, based on the results of this study, we cannot exclude the direct effect of SMe1EC2M3 on other molecular targets in addition to the SERT, NET, and DAT. Other pharmacological studies, such as receptor binding and/or microdialysis assessments, as well as behavioral studies after chronic treatment with SMe1EC2M3, should be performed to complete the pharmacological profiling of this compound.

## 4. Materials and Methods

### 4.1. In Silico Study

We used Dragon 6.0 software (Talete srl, Milan, Italy) for the prediction of antidepressant, antipsychotic, and hypnotic activity of SMe1EC2M3. Dragon enabled us to calculate several thousands of molecular descriptors, which can be further used for QSAR study. Using the Comprehensive Medicinal Chemistry (CMC) and Available Chemicals Directory (ACD) databases, Ghose et al. [40] derived preferred ranges of the values of calculated octanol-water partition coefficient (SKlogP), molar refractivity and molecular weight for the molecules with antidepressant, antipsychotic or hypnotic activity. Authors worked on the assumption that simple and reliably predictable properties of molecules as the partition coefficient (representing a transport and bioavailability), molar refractivity (proportional to the polarizability of molecule and thus reflecting its binding to proteins), molecular weight together with the number of atoms (two of the basic parameters for Lipinski’s rule of five for a drug likeness) could represent a proper choice to evaluate sufficiently large set of compounds. The corresponding descriptors Depressant-50, Psychotic-50 and Hypnotic-50 for possessing the individual activities were directly estimated by Dragon. The meaning of “-50” suffix was derived from authors decision to choose 50% from the set of active compounds near the maximum of the Gauss population curve and setting the limits for this interval. In addition, fish baseline toxicity factor-96 (BLTF96), calculated by the same software, was taken as an assessment of the toxicity [17]. Software PreADMET (Version 2.0, [41]) was used for the estimation of blood-brain barrier penetration (BBB), human intestinal absorption (HIA), plasma protein binding (PPB), as well as the SKlogP and octanol/water distribution coefficient (SKlogD) at pH = 7.4.

### 4.2. In Vivo Studies

#### 4.2.1. Animals

Male Wistar rats (weighting 220–240 g, 3 months of age) were used in the study. The rats were obtained from the Department of Toxicology and Laboratory Animal Breeding of the Institute of Experimental Pharmacology and Toxicology, Centre of Experimental Medicine, Slovak Academy of Sciences (Dobrá Voda, Slovakia). Animals were housed under standard laboratory conditions (temperature: 22 ± 2 °C, humidity: 55 ± 10%) with a 12 h light/12 h dark cycle (lights on at 7 a.m.). Pelleted food and tap water was available ad libitum. All experimental procedures were approved by the Animal Health and Animal Welfare Division of the State Veterinary and Food Administration of the Slovak Republic (Permit number Ro 3054/17-221/3) and conformed to the Directive 2010/63/EU of the European Parliament and of the Council on the Protection of Animals Used for Scientific Purposes.

#### 4.2.2. Drugs

SMe1EC2M3 was synthetized in the Faculty of Natural Sciences, Comenius University in Bratislava, Slovakia, and dissolved in sterile water for injection (vehicle). Purity of the compound was greater than 95% as determined by ^1^H-NMR analysis. For the behavioral experiments, SMe1EC2M3 was administered intraperitoneally (i.p.) at the doses of 5 and 25 mg/kg. Control animals were administered vehicle. For the electrophysiological experiments, SMe1EC2M3 was administered intravenously (i.v., via a catheter placed in the lateral tail vein) at the doses 0.5–2.5 mg/kg. The doses were chosen according to our previous studies with pyridoindole derivatives [42,43]. Chloral hydrate, WAY100135, and yohimbine were purchased from Lambda Life, a.s. (Bratislava, Slovakia) and dissolved in vehicle. Injectable solution of haloperidol was purchased from Gedeon Richter Plc. (Budapest, Hungary).

#### 4.2.3. Behavioral Experiments

Behavioral assessments were performed as previously described [21]. Shortly, rats were acclimatized to the animal housing facility for two weeks prior to experimental procedures. The movement of the rat was tracked with a digital camera and analyzed by computer software ANYMAZE™ (Stoelting Europe, Dublin, Ireland). All experiments were conducted during the light phase; between 8 a.m. and 1 p.m. Assignment of animals into experimental groups was conducted prior to experimental procedures based on their basal motor activity in open field test (dark arena size of 60 × 60 cm with ambient light). Each session started by placing the rat in the central area of the maze and let freely explore for five minutes. After obtaining the data of distance travelled in open field test, we randomized these values and subsequently used one-way ANOVA test to prove the means between groups do not have a difference.

For the FST, the rats were placed in a glass cylinder (45 cm tall and 25 cm in diameter) filled with water (24 ± 1 °C) for a 15 min pre-test period to induce depression-like behavior. The following five min test started 24 h later. The depth of the water (30 cm) was sufficient to ensure that the animals could not touch the bottom of the container with their hind paws. SMe1EC2M3 was administered immediately after the pre-test and one hour before the test [44]. The animals were returned to their home cage after resting under a heating lamp until dry. The time of immobility, climbing and swimming of each animal was scored manually. Immobility (passive behavior) was defined as floating behavior without any movements other than those necessary to balance the body and keep the head above the water [25]. Escape-directed (active) behaviors were scored separately as vertical movement of the forepaws (climbing) or horizontal movement throughout the swim chamber (swimming).

#### 4.2.4. Electrophysiological Experiments

In vivo electrophysiological experiments were performed as described in our previous study [32]. Experiments were performed in separate group of animals not used for behavioral studies. Animals were anesthetized by chloral hydrate (400 mg/kg, i.p.) and mounted in the stereotaxic frame (David Kopf Instruments, Tujunga, CA, USA). Rat body temperature was maintained between 36–37 °C with a heating pad (Gaymor Instruments, Orchard Park, NY, USA). The scalp was opened and a 3 mm hole was drilled in the skull for insertion of electrodes. Glass-pipettes were pulled with a DMZ-Universal Puller (Zeitz-Instruments GmbH, Martinsried, Germany) to a fine tip approximately 1 μm in diameter and filled with 2M NaCl solution. Electrode impedance ranged from 4 to 6 MΩ. The pipettes were lowered into the target brain area by hydraulic micro-positioner (David Kopf Instruments). Signals were amplified by HEKA EPC-10 amplifier and recorded by Pathmaster software package (HEKA Elektronik-Dr Schulze GmbH, Lambrecht/Pfalz, Germany).

Serotonin neurons were recorded from the dorsal raphe nucleus (DRN), 7.8–8.3 mm posterior to bregma and 4.5–7.0 mm ventral to brain surface [45]. The 5-HT neurons were identified by their regular firing rate of 0.5–2.5 Hz and positive action potential of long duration of 0.8–1.2 msec, as described in our previous studies [32,46,47]. After a 5-HT neurons was identified and its basal firing activity was recorded for 2 min, SMe1EC2M3 was administered intravenously at the cumulative doses of 0.25–1.50 mg/kg (i.v). After the last SMe1EC2M3 exposure, an antagonist of 5-HT_1A_ receptors, WAY100135 administered at the dose of 0.1 mg/kg (i.v.), as previously described [33]. Minimal two-minute interval was preserved between the administration of the different doses of SMe1EC2M3 and between the last dose of SMe1EC2M3 and WAY100135.

Norepinephrine neurons were recorded from the locus coeruleus (LC), 8.0–8.3 mm posterior to bregma, 1.2–1.4 mm lateral to the midline, and 5.5–7.5 mm ventral to the brain surface [26]. Norepinephrine neurons were recognized by regular firing rate of 0.5–5.0 Hz, positive action potential of long duration of 0.8–1.2 msec and a characteristic burst discharge in response to nociceptive pinch of the contralateral hind paw, as described in our previous studies [32,46,47,48]. After a norepinephrine neuron was identified and its basal firing activity was recorded for 2 min, SMe1EC2M3 was administered intravenously (i.v.) at the cumulative doses of 0.50–2.50 mg/kg. After the last SMe1EC2M3 exposure, an antagonist of α_2_-adrenoreceptors, yohimbine, was administered at the dose of 0.1 mg/kg (i.v.), as previously described. Minimal two-minute interval was preserved between the administration of the different doses of SMe1EC2M3 and between the last dose of SMe1EC2M3 and yohimbine.

Dopamine neurons were recorded from the ventral tegmental area (VTA), 4.5–5.5 mm posterior to bregma, 0.6–0.8 mm lateral to the midline, and 7.0–8.5 mm ventral to the brain surface [45]. Dopamine neurons were recognized by their slow irregular firing-rate of 0.5–10 Hz, mixed single-spike and burst firing, tri-phasic action potentials with a dominant positive component, a minor one with duration over 2.5 msec. and a “notch” often present on the initial rising phase and a minimum 1.1 msec duration from action potential initiation to the negative trough, as described in our previous studies [32,47,48,49,50,51,52,53]. After a dopamine neuron was identified and its basal firing activity was recorded for 2 min, SMe1EC2M3 was administered intravenously (i.v.) at the cumulative doses of 0.50–2.50 mg/kg. After the last SMe1EC2M3 exposure, an antagonist of D_2_-adrenoreceptors, haloperidol, was administered at the dose of 0.1 mg/kg (i.v.), as previously described. Minimal two-minute interval was preserved between the administration of the different doses of SMe1EC2M3 and between the last dose of SMe1EC2M3 and haloperidol.

In electrophysiological experiments, the initial dose of SMe1EC2M3 (0.25 mg/kg for 5-HT neurons and 0.5 mg/kg for norepinephrine and dopamine neurons) was chosen according to the previous electrophysiological studies with other TRIs SEP-225289 and DOV216303 [10]. Additional doses of SMe1EC2M3 were applied until the maximal inhibition of neuronal excitability was observed (5-HT neurons: cumulative dose of 1.5 mg/kg; norepinephrine and dopamine neurons: cumulative dose of 2.5 mg/kg).

### 4.3. Statistical Analysis

For the behavioral experiments, all data was presented as mean ± Standard Error of the Mean (SEM). One-way analysis of variance (ANOVA), followed by the Fisher´s LSD post-hoc test, was used to evaluate the effect of SMe1EC2M3 on animal behavior during the FST. For the electrophysiological experiments, the excitability of 5-HT, norepinephrine, or dopamine neurons after the administration of SMe1EC2M3 and WAY100135, yohimbine, or haloperidol was expressed as % ± SEM of baseline. One-way ANOVA for repeated measures, followed by Fisher´s LSD post-hoc test, was used to compare the firing activity of 5-HT, norepinephrine, and dopamine neurons after SMe1EC2M3 and WAY100135, yohimbine, or haloperidol administration, with the baseline.

## Figures and Tables

**Table 1:** In silico prediction of pharmacokinetics and pharmacodynamics of SMe1EC2M3. SMe1EC2M3, ((±)-*cis* ethyl 8-methoxy-6-methyl-3,4,4a,5,9b*H*-hexahydro-1*H*-pyrido[4,3-b]indole-2-carboxylate; BBB, blood-brain barrier; C_brain_/C_blood_, brain/blood concentrations; SKlogD, octanol/water distribution coefficient; SKlogP, octanol-water partition coefficient; BLTF96, fish baseline toxicity factor-96; the expectancy of anti-depressant (anti-depressant-50), anti-psychotic (anti-psychotic-50) and hypnotic properties (hypnotic-50).

Predicted Property	Value	Source
BBB permeability (C_brain_/C_blood_)	0.13	PreADMET
Human Intestinal Absorption (%)	95.37	
Plasma Protein Binding (%)	80.97	
SKlogD	2.37	
SKlogP	2.37	
BLTF96 (mmol/L)	−2.85	Dragon
anti-depressant-50*	Yes	
anti-psychotic-50*	No	
hypnotic-50*	No	

* For the explanation of the parameters see Methods.
